# Patients with early-onset systemic juvenile idiopathic arthritis show more inflammation and worse outcome

**DOI:** 10.1186/1546-0096-9-S1-O18

**Published:** 2011-09-14

**Authors:** R Russo, M Katsicas

**Affiliations:** 1 Service of Immunology & Rheumatology, Hospital de Pediatría Prof. Dr. Juan P. Garrahan, Buenos Aires, Argentina

## Background

Systemic Juvenile Idiopathic Arthritis (SJIA) is heterogeneous in severity, course and outcome. Predictive factors for a poor outcome include persistent systemic features (fever, thrombocytosis) and younger age at onset.

## Aim

To describe and analyze the disease features in patients with SJIA with very early onset and to compare them with those of patients with later onset.

## Methods

Retrospective analysis of clinical data. Early-onset (EO) was defined as the start of SJIA prior to age 18 months. Variables included: demographic and clinical features at onset and outcome variables during disease course (pattern of course, presence of clinical joint damage [using the Juvenile Arthritis Damage Index or JADI], radiographic joint damage [erosions], destructive hip disease, disability [CHAQ > 0.5], growth retardation, development of macrophage activation syndrome [MAS], need for biologic agents, and death). Patients with EO were compared with patients with disease onset at age ≥ 18 months. Chi square and Mann-Whitney tests were used for comparisons.

## Results

192 patients (115 girls, 23 EO) followed between 1995 and 2010 were included. Age at onset was 12 (2-17) months in patients with EO, 72 (18-191) months in patients with non-EO. Delay in diagnosis (2 months) and duration of follow-up (10 vs 8 years) were similar in both groups. Eight patients (1 EO, 7 non-EO) died during the observation period. Patients with EO showed more frequently serositis (p=0.0003), hepatomegaly (p=0.01), splenomegaly (p=0.03), lower number of active joints (p=0.01), Hgb (p=0.02), and higher plt (p=0.04) at onset; more frequently MAS (p=0.0001), therapy with biologics (p=0.01), destructive hip disease (p=0.04), radiographic damage (p=0.01), growth retardation (p=0.05), disability (p=0.01), and higher JADI score (p=0.003).

## Conclusions

Patients with SJIA starting before age 18 months show more systemic inflammatory features, and a poorer outcome than children with later disease onset. They may represent a distinct subset. This observation should prompt rheumatologists to initiate early aggressive therapy and close follow-up in this age group.

